# Hospital Administration

**Published:** 1898-07-16

**Authors:** 


### THE INSTITUTIONAL WORKSHOP.

### HOSPITAL ADMINISTRATION.

### ADDENBROOKE'S HOSPITAL, CAMBRIDGE.

The Special Finance Committee of this hospital, appointed by the Governors to inquire into the management and finances of the institution, submitted the following report to the last General Quarterly Court: "The Special Finance Committee have, in accordance with the instructions of the Quarterly Court, consulted Sir Henry Burdett as an expert in hospital management. He has prepared a report giving the result of his inquiries, and making a number of important recommendations as to the administration, &c., of the hospital. This report the committee desire to lose no time in laying before the Governors, and accordingly submit it to the court. They recommend that a copy be sent to each of the Governors, and that the court at its rising adjourn for one month in order that the report may be carefully considered in the meantime, and that at the adjourned court order may be made as to the steps to be taken in reference thereto." After some discussion it was resolved that a committee, consisting of the Special Finance Committee, who should have power to co-opt not more than three additional members, should be at once appointed, and bring their recommendations before the Michaelmas Quarterly Court.

SIR HENRY BURDETT'S REPORT.

We take the following abstract of the report on certain proposed changes in the constitutional, financial, nursing, administrative, and structural arrangements of Addenbrooke's Hospital from the *Cambridge Independent*:—

"Sir Henry deals first with the matter of staff appointments. He recommends that a Selection Committee, for the purpose of appointing the honorary medical officers of Addenbrooke's Hospital, be set up at the first General Court in each year, and that it consist of 50 Governors, 18 of whom shall be the members of the Committee of Management hereinafter referred to, 8 the honorary physicians and surgeons for the time being, and 24 other Governors, namely, eight from each of the three groups of Governors who are recognised by their *ex officio* representation on the governing body, in terms of the Special Act of Parliament. The Act of Parliament provides that the county is to be represented by the Lord Lieutenant, the High Sheriff, and the Members of Parliament for the county; the borough is to be represented by the Mayor, the High Steward, and the member of Parliament for the borough; and the University by the Chancellor, the Vice-Chancellor, and the University Members of Parliament. He therefore recommended that three groups of eight Governors shall be elected from the Governors, by ballot or otherwise, to represent the county, the borough, and the University respectively, making collectively 24 special Governors.

"He adds: I have been led to recommend the appointment of this Special Selection Committee because I am familiar with the abuses that might result from the present system of vesting the election in the whole body of Governors. I would suggest that a new bye-law should be passed, providing that any candidate who directly or indirectly canvasses the Governors should be disqualified. I am further of opinion that it is desirable to modernise the constitution of the hospital by the introduction of the following additional modifications. In lieu of the open Weekly Board of Governors which prevails, and which any Governors who please may attend and vote at, I strongly recommend the appointment of a Committee of Management, consisting of, say, eighteen lay Governors, with some or all of the physicians and surgeons who collectively number eight; the Committee of Management to be elected at a general court, and one-third of its members to retire every year, being eligible for re-election. Out of this General Committee of Management I recommend that an Executive Committee of seven Governors and a Finance Committee of five Governors should be elected by the members of the General Committee, each of which committees should be summoned weekly to transact the executive and financial business of the institution.

"It is, moreover, of the first importance that your institution should have the services of an active governor as chairman of the Committee of Management, who should have authority to decide, between the sittings of the Committee of Management, any matter which pressed for settlement.

"These changes in its constitution, if made, would, by increasing the responsibility and the controlling power of the executive bodies, tend materially to improve the administration of the institution and the systematising of its business, and should at the same time stimulate the public interest and confidence in Addenbrooke's Hospital, its welfare and progress.

"ECONOMY, FINANCE, AND ACCOUNTS.

"Economy has been compared to a cone which should properly stand upon its base, though some people are liable to spend much time in endeavouring to balance it upon its apex. I mention this because I have followed for some years the proceedings of the Governors of Addenbrooke's Hospital, and from the discussions that have arisen I have come to the conclusion that there is abroad a very general impression that this institution has been extravagantly conducted. I, therefore directed my inquiries in the first place to this point of economy, and I endeavoured to find out, if it were possible, the causes which have lead to this impression amongst certain of the Governors and others. After a most exhaustive series of tests, I am glad to be able to assure the Governors that if they err at all they err, and have erred for some years, not in the direction of over-expenditure, but of under-expenditure. In conducting the affairs of a public institution, and especially of a hospital, it is possible to be too economical, having regard to the important interests which have to be conserved. The tables attached to this report will enable anyone who is interested to compare the expenditure under various headings at different hospitals with that of Addenbrooke's Hospital. It will be found that on the whole Addenbrooke's Hospital comes very favourably out of this comparison, a fact which is not to be wondered at when it is known that the cost of provisions at Addenbrooke's Hospital, including the board of both patients and staff, was in 1894-5 6s. 4d., in 1895-6 6s. 1d., and in 1896-7,

"6s. 2d. PER HEAD PER WEEK.

Of course, there are differences between the several hospitals, but these differences can be explained without in any way reflecting upon the economical administration of Addenbrooke's Hospital. Thus the increase in expenditure, comparing 1894-5 with 1896-7, amounts to about £423, and is mainly accounted for by the increase in the staff during the year 1896-7 by the addition of eight ward maids, and by two additional beds having been constantly occupied during 1896-7. This increase is not a great matter, and it would probably have coincided with increases in the other institutions had the accounts of Addenbrooke's Hospital been kept on the 'Uniform System.' I am led to this conclusion by the circumstance that at the Queen's Hospital, where the cost per occupied bed is about the same or rather less than it is at Addenbrooke's, I find that the number of beds constantly occupied is greater than at Addenbrooke's, a fact which more than accounts for any difference of this kind, when it is known that the Queen's Hospital accounts are kept on the Uniform System, and that every item is therefore accurately subdivided and credited to its proper head. This is not the case at Addenbrooke's, and I therefore recommend that the Uniform System of accounts should be introduced, as that will probably prove the best safeguard to economical administration which it is within the power of the Governors to introduce. I have further to point out that the

"SYSTEM OF FINANCING THE HOSPITAL

at the present time, which has been settled by order of the Governor, does not commend itself to my judgment as a man of business. Every year it has been the practice to bring forward a balance of varying amount, as due to the treasurer, instead of providing that each year's accounts shall be closed, and any outstanding balance to the treasurer paid off. It is very misleading to bring forward this balance year after year, a fact which will be recognised by all when I point out that the balance brought forward for the year ended Michaelmas, 1885, was £836, and that this balance, after fluctuating in intervening years, amounted at Michaelmas, 1897, to £1,521. Notwithstanding this circumstance, the invested property, which amounted in 1885 to £47,870, had increased in 1897 to £51,536, including £750 transferred to the Samaritan Fund, although £4,775 stock had been sold and the proceeds expended during the period mentioned. It is now generally admitted that any hospital which has

"Invested Property to the extent of five times its gross annual expenditure may properly consider any invested funds beyond that amount as a reserve available for the purposes of extension, reconstruction, or new buildings, at the discretion of the managers for the time being. Assuming, therefore, that the outside gross expenditure of Addenbrooke's Hospital—if my recommendations are adopted—will in future never exceed £8,500 per annum, the hospital has a reserve fund of nearly £8,500 available for the improvement or extension of its buildings, as may be found desirable. And here I would bear testimony to the obligation which the Governors are under to Mr. Edmund John Mortlock, M.A., the treasurer, who has given the services of one clerk and has every year lent, without interest, to the general account of the hospital sums varying up to £1,500, according to the overdraft for the time, from the earliest year of which I have investigated the accounts, namely, 1885. The clerk's services with the interest on the average overdraft must have represented a gift of something like £150 per annum, a fact which I beg the Governors to bear in mind in considering my recommendations as to the appointment of a resident superintendent, a part of whose duty it will be to relieve the treasurer of all clerical duties by undertaking to keep the books at the hospital under the treasurer's direction. The present system of

"Investing all Legacies as and when received is not the best, and is neither economical nor desirable. I would suggest that all legacies as received should be placed to deposit account at the bankers' at interest, and that the investment of such legacies, or the sale of stock, should be settled at the end of each financial year by the Committee of Management on the recommendation of the treasurer and Finance Committee. Turning now to the income side of the account, I find that the income of the hospital from all sources, including legacies, has exceeded the total ordinary and extraordinary expenditure during each of the three years 1894-5 to 1896-7, a fact which shows that the public are prepared to contribute all the money necessary to maintain Addenbrooke's Hospital in a high state of efficiency. I think, however, that it might be well that an effort should be made to raise the total income to £10,000 per annum. Finally, it is perfectly clear to my mind, from a study of the figures, that any apparent differences in the expenditure on particular items are largely due to the non-adoption of the Uniform System of accounts. This system would have secured a precise analysis of the expenditure, and at the same time have enabled a careful check to be exercised over each item of expenditure from month to month.

"Nursing.

"I notice that, whereas a very considerable income (£875 on an average of the last three years), is received from probationers' fees, your hospital has not heretofore provided the probationers with uniform or paid them a salary when they are promoted to staff duty in their third year. I would strongly recommend that a salary of, say, £20 should henceforth be paid to every probationer who in her third year may be appointed a staff nurse, such a step being in accordance with the practice now adopted by the best-managed hospitals throughout the country. I would further impress upon the Governors the fact that they have been most fortunate in securing a high class of women, who have given their services, nay, who have paid considerable sums, in order to be trained as nurses at Addenbrooke's Hospital. These ladies have shown great forbearance in consenting for many years to carry out duties which ought to have been relegated to ward maids, and I congratulate the Governors upon the wisdom which they have displayed in introducing ward maids into the hospital during the last year. I would, however, impress upon them the desirability of lessening the hours of duty of the probationer-nurses, which at the present time are excessive. Indeed, the hours and duties are more arduous than those of some of the best conducted hospitals in the country.

"General.

"Generally I may say that in visiting the hospital I have often been struck by the unattractive appearance of the corridors and passages, and especially by the untidy and neglected condition of the open balconies of the hospital. These balconies were intended no doubt by the architect to be used as places where the patients could be placed so as to get the maximum of air and sunlight whenever the weather admitted of such treatment. At the present time they often contain clothes-horses, ashbins, and lumber of various kinds; the breast-walls are so high as to prevent the patients from looking out if they are placed in beds, as they might otherwise be; and there is an absence of proper and comfortable seats where an invalid might recline or sit with advantage when permitted to do so by the doctors. These defects could easily be remedied by the expenditure of a little money upon paint, internal decoration, and furniture. The patients could readily be enabled to look out either by raising the present floors of the balconies, or by substituting an ornamental iron railing for a portion of the present brickwork. I should strongly urge the importance of immediate attention being given to this matter, because the present state of the passages, staircases, corridors, and balconies, when compared with the brightness of the wards, gives, I am sure, a false impression to the visitor, who might be excused if, in existing circumstances, he gathered the impression that the administration of the hospital was not so satisfactory as it ought to be.

"General Recommendations.

"Having gone carefully over the whole administration, and fully considered the various points of detail which were gone into when I had the pleasure of meeting the Special Finance Committee, I am confident that it would tend to promote economy, to increase the income, and to place Addenbrooke's Hospital administratively in a strong position, if the Governors were to decide to elect a resident superintendent of the hospital at a salary of, say, £250 per annum, with board and residence. He should be charged with the general administration of the institution, the supervision and purchase of the stores, and the general discipline of the household so far as the male servants are concerned. Such an officer should further be entrusted with the duty of introducing the Uniform System of accounts, seeing that at the present time it is impossible to ascertain accurately and in sufficient detail to what extent, and for what reason, certain items of expenditure have increased in recent years. It is not possible to state authoritatively where, if at all, increases have occurred, because the books have not hitherto been kept on this system, and analytical details are wanting. Such an appointment and the introduction of the changes recommended would speedily, Sir Henry thinks, bring about an increase in public confidence and support, which should ere long raise the income of the hospital to £10,000 a year, or whatever sum may be needed to enable the committee and the medical staff to maintain the hospital in the highest state of efficiency with due regard to the maximum of economy."

